# Immobilization of *Aspergillus oryzae* *β*-Galactosidase on Cellulose Acetate-Polymethylmethacrylate Membrane and Its Application in Hydrolysis of Lactose from Milk and Whey

**DOI:** 10.1155/2014/163987

**Published:** 2014-10-28

**Authors:** Shakeel Ahmed Ansari, Rukhsana Satar, Syed Kashif Zaidi, Abrar Ahmad

**Affiliations:** ^1^Center of Excellence in Genomic and Medicine Research, King Abdulaziz University, Jeddah 21589, Saudi Arabia; ^2^Department of Biochemistry, Ibn Sina National College for Medical Sciences, Jeddah 21418, Saudi Arabia; ^3^Environmental Biotechnology Division, CSIR-Indian Institute of Toxicology Research, M.G. Marg, Lucknow 226001, India

## Abstract

The present study demonstrates the immobilization of* Aspergillus oryzae β*-galactosidase on cellulose acetate-polymethylmethacrylate (CA-PMMA) membrane and its application in hydrolyzing lactose in dairy industries. The effect of physical and chemical denaturants like pH, temperature, product inhibition by galactose, storage stability, and reuse number of the enzyme immobilized on CA-PMMA membrane has been investigated. Lactose was hydrolyzed from milk and whey in batch reactors at 50°C by free and immobilized *β*-galactosidase (I*β*G). Optimum pH for the free and immobilized enzyme was found to be the same, that is, 4.5. However, I*β*G retained greater fractions of catalytic activity at lower and higher pH ranges. The temperature optimum for the immobilized enzyme was increased by 10°C. Moreover, Michaelis-Menten constant was increased for I*β*G as compared to the native one while maximum reaction rate was reduced for the immobilized enzyme. The preserved activity of free and immobilized enzyme was found to be 45% and 83%, respectively, after five weeks of storage at 4°C. Reusability of I*β*G was observed to be 86% even after fifth repeated use, thereby signifying its application in lactose hydrolysis (as shown in lab-scale batch reactors) in various dairy products including milk and whey.

## 1. Introduction

Recent years have witnessed the modification of polymers for improving their physical and mechanical properties so as to utilize them in several industrial applications. These include cellulose acetate (CA), polysulfone, polymethylmethacrylate (PMMA), polystyrene, and polycarbonate. Moreover, the utilization of membranes has attracted the attention of the enzymologist for applying them as highly efficient and stable support in immobilizing industrially important enzymes [1–3].

CA has gained the attention of the researchers in the recent past due to its hydrophilicity, low cost, selective modification, excellent fouling resistance, and availability in various grades. However, major disadvantages associated with it include low oxidation and chemical resistance and poor mechanical strength [4]. On the other hand, PMMA is glassy polymer that possesses fine mechanical, thermal, and optical properties. Major drawback associated with its use is its greater hydrophobicity which leads to reduction of flux. Hence, in order to apply PMMA membrane efficiently in industrial applications, its hydrophilicity is improved by blending it with CA [5].

Needless to mention, enzyme immobilization is an important process for facilitating the continuous and long-term processing of the biocatalyst [6]. Numerous carriers and technologies have been implemented by researchers for improving the immobilization of enzyme in order to enhance their activity and stability to decrease the enzyme biocatalyst cost in industrial biotechnology. These include cross-linked enzyme aggregates, microwave-assisted immobilization, click chemistry technology, recombinant enzymes, and nanoparticle-based immobilization of enzymes [7]. Therefore, in the present study, CA was blended with PMMA to superimpose requisite properties and maintain the hydrophilic-hydrophobic balance of the developed membrane system.

*β*-Galactosidase (EC 3.2.1.23) catalyzes the hydrolysis of lactose into glucose and galactose. It is found in plants, microorganisms, and animals and is widely used in food industry due to its hydrolytic activity on lactose and transferase activity on galacto-oligosaccharides production [8, 9]. The membranes which were used for immobilizing *β*-galactosidase include polyvinyl chloride microspheres [10], nylon membranes [11], polyvinylidene fluoride membrane [12], and cellulose acetate membranes [13] for providing galacto-oligosaccharides while polyethersulfone membrane was utilized for immobilizing* Kluyveromyces fragilisβ*-galactosidase to hydrolyze whey lactose in dairy industries [14]. An excellent review has appeared lately in which *β*-galactosidase from mesophilic, psychrophilic, and thermophilic organisms was utilized for obtaining galacto-oligosaccharides and lactose-free dairy products [15].

Hence, in this study, a simple, efficient, and inexpensive procedure has been developed to immobilize* Aspergillus oryzae* *β*-galactosidase on CA-PMMA membrane. Effect of various physical and chemical denaturants on the activity of soluble *β*-galactosidase (S*β*G) and CA-PMMA adsorbed *β*-galactosidase (I*β*G) has been investigated. Effect of product inhibition by galactose has also been evaluated for studying its potential biotechnological application in lactose hydrolysis. Soluble and immobilized enzyme has been evaluated for the hydrolysis of lactose from milk and whey in batch process at 50°C.

## 2. Experimental

### 2.1. Materials and Methods

*β*-Galactosidase from* Aspergillus oryzae*, polymethylmethacrylate, cellulose acetate, glucose oxidase-peroxidase assay kit, and* o*-nitrophenyl *β*-D-galactopyranoside (ONPG) was obtained from Sigma Chem. Co. (USA). Glutaraldehyde was purchased from Thomas Baker Chemical Co. (India). Milk and whey were purchased from local market. Other chemicals and reagents employed in the study were of analytical grade and used without any further purification.

### 2.2. Preparation of Cellulose Acetate-Polymethylmethacrylate (CA-PMMA) and Its Characterization by Scanning Electron Microscopy

Cellulose acetate-polymethylmethacrylate (CA-PMMA) membrane was prepared with slight modification according to the procedure described by Rauf et al., 2006 [16]. This method involves the dissolving of 0.7 g cellulose acetate and 0.2 g polymethylmethacrylate in 10 mL of acetone-chloroform mixture (4 : 1). The resulting solution was spread on a glass slide having thickness of 75 × 25 mm. The solvent was allowed to evaporate for half an hour and the thickness of membrane was adjusted to 1.0 mm with the help of a spreader. Analysis of the surface and cross-section of freeze dried samples of CA-PMMA was performed with Philips-515 scanning electron microscope (U.S.A.). The membrane samples were mounted on an aluminum sample mount and sputter coated with gold to minimize surface charging. The specimens were observed at a 15 kV accelerating voltage.

### 2.3. Activation of  CA-PMMA

CA-PMMA membranes were activated by 2.0% glutaraldehyde solution for 2 hours followed by washing with deionized water thrice to remove excess of glutaraldehyde. The membranes got activated by glutaraldehyde as a result of adsorption phenomenon which provides a highly efficient matrix for immobilization of *β*-galactosidase.

### 2.4. Immobilization of *β*-Galactosidase on CA-PMMA Membranes

CA-PMMA membrane was placed in *β*-galactosidase (15000 U) solution prepared in 100 mM sodium acetate buffer pH 4.5 at 4°C for 24 h and then washed thoroughly with deionized water to remove the unbound enzyme. The membranes were finally cut into 6.45 cm^2^ pieces at the end of immobilization.

### 2.5. Assay of *β*-Galactosidase

The hydrolyzing activity of *β*-galactosidase was determined by measuring the release of* o*-nitrophenol from* o*-nitrophenyl *β*-D-galactopyranoside at 405 nm. The reaction was carried out with continuous shaking in an assay volume of 2.0 mL containing 1.7 mL of 100 mM sodium acetate buffer, pH 4.5, 0.1 mL *β*-galactosidase (2.0 U), and 0.2 mL of 20 mM ONPG. The reaction was stopped by adding 2.0 mL sodium carbonate solution (1.0 M) and* o*-nitrophenol formation was measured spectrophotometrically at 405 nm [9].

One unit (1.0 U) of *β*-galactosidase activity is defined as the amount of enzyme that liberates 1.0 *μ*mole of *o*-nitrophenol (*ε*_*m*_ = 4500 L/mol/cm) per min under standard assay conditions.

### 2.6. Determination of Kinetic Parameters

Lineweaver Burk plot was used to measure Michaelis-Menten constant and maximum reaction rate at varying concentrations of ONPG in 100 mM sodium acetate buffer at pH 4.5.

### 2.7. Effect of pH and Temperature

Enzyme activity (2.0 U) of S*β*G and I*β*G was assayed in 100 mM buffers of various pH ranges (pH 3.0–9.0). The buffers used were glycine-HCl (pH 3.0), sodium acetate (pH 4.0, 4.5, and 5.0), sodium phosphate (6.0-7.0), and Tris-HCl (pH 8.0-9.0). The activity at pH 4.5 was taken as control (100%) for calculating the remaining percent activity at other pH ranges.

Similarly, the effect of temperature was observed by measuring the activity of S*β*G and I*β*G (2.0 U) at various temperatures (30–80°C). The enzyme activity obtained at 50°C was taken as control (100%) for the calculation of remaining percent activity.

### 2.8. Effect of Galactose

The effect of various concentrations of galactose (1.0–5.0%, w/v) on the activity of S*β*G and I*β*G (2.0 U) was measured independently in 100 mM sodium acetate buffer pH 4.5 at 50°C. The activity of enzyme without added galactose was considered as control (100%) for calculating the remaining percent activity.

### 2.9. Storage Stability and Reusability

S*β*G and I*β*G were stored at 4°C in 100 mM sodium acetate buffer pH 4.5 for 6 weeks. The aliquots from each preparation (20 *μ*L) were taken in triplicates every week and analyzed for the remaining activity. The activity determined on the first day was taken as control (100%) for the calculation of remaining percent activity.

Reusability of I*β*G (20 *μ*L) was taken in triplicates for assaying the activity of enzyme. After each assay, immobilized enzyme was taken from assay tubes and stored in 100 mM sodium acetate buffer pH 4.5 overnight at 4°C for 6 successive days. The activity determined on the first day was considered as control (100%) for the calculation of remaining percent activity.

### 2.10. Lactose Hydrolysis from Milk and Whey in Batch Process

Milk and whey (200 mL) were independently incubated with S*β*G and I*β*G (100 U) in water bath at 50°C for various time intervals and stirred continuously. The aliquots were taken at different times and assayed for the formation of glucose by glucose oxidase-peroxidase assay kit.

### 2.11. Estimation of Protein

Protein concentration was determined according to the procedure described by Lowry et al., 1951 [17]. Bovine serum albumin was used as a standard.

### 2.12. Statistical Analysis

Every experiment was performed in triplicates with average standard deviations <5%. Data expressed in the study was plotted by using Sigma Plot-9. Data was analyzed by one-way ANOVA. *P* values <0.05 were considered statistically significant.

## 3. Results and Discussion

Immobilized enzymes have numerous biomedical and industrial applications which continued their development into an ever-expanding and multidisciplinary field during the last two decades [18, 19]. Henceforth, new strategies are also continuously emerging to immobilize *β*-galactosidase for producing lactose-free dairy products in dairy industries. Several researchers have previously utilized cellulose acetate and polymethylmethacrylate independently for immobilizing *β*-galactosidase previously [13, 19, 20]. However, major problem associated with them involves the mass transfer phenomenon of substrates, reaction products, and inhibitors through the membrane as well as with microorganisms growing on the membrane surface which leads to the inhibition of enzyme layer and ultimately making the behavior of immobilization matrix poorly.

### 3.1. Characterization of CA-PMMA Membrane and Its Utility in Immobilizing *β*-Galactosidase

**Figure 1:**
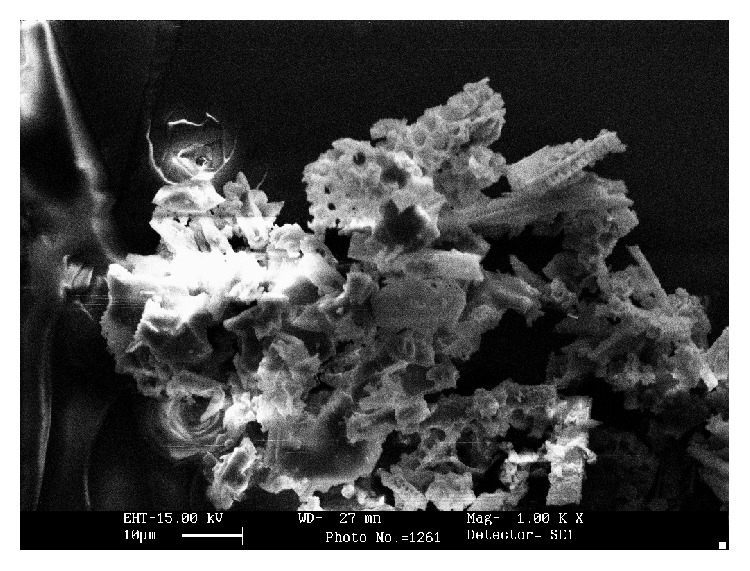
Scanning electron micrographs of CA-PMMA. The interaction of CA with PMMA was monitored with Philips-515 scanning electron microscope by mounting them on an aluminum sample and sputter coating them with gold to minimize surface charging. The specimens were observed at a 15 kV accelerating voltage.

The present study involves the blending of CA with PMMA to superimpose requisite properties and maintain the hydrophilic-hydrophobic balance of the developed membrane system (Figure 1). The developed CA-PMMA membrane was exploited for immobilizing* Aspergillus oryzae* *β*-galactosidase (93%) for increasing its stability and efficiency in producing lactose-free dairy products in dairy industries (Table 1).

### 3.2. Kinetic Parameters

The data obtained from Lineweaver Burk plot suggested that Michaelis-Menten constant was increased while maximum reaction rate was decreased as a result of immobilization (Table 2). Mass transfer resistance and electrostatic and steric effects might be the probable reason for an increase in Michaelis-Menten constant obtained after immobilization [21, 22]. It should be noted that mass transfer resistance appeared significant for macromolecular substrates such as ONPG because the substrate must contact the enzyme adsorbed on the surface of CA-PMMA. Moreover, immobilization resulted in less accessibility of enzyme active sites to the substrate than the free enzyme. Additionally, immobilization of *β*-galactosidase on CA-PMMA might have reduced its ability to undergo conformational changes that are intrinsic to enzyme-substrate interaction, thereby decreasing maximum reaction rate for the immobilized enzyme. The increase in Michaelis-Menten constant and decrease in maximum reaction rate for the immobilized enzyme seen here are in agreement with* Kluyveromyces lactis* *β*-galactosidase immobilized on modified carbon nanotubes [23].

### 3.3. Effect of Physical and Chemical Denaturants

**Figure 2:**
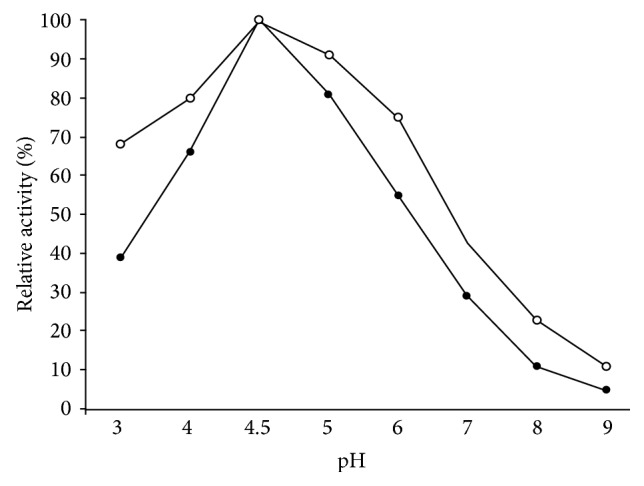
pH activity profiles for soluble and CA-PMMA bound *β*-galactosidase. Activity of soluble and immobilized *β*-galactosidase (20 *μ*L) was measured at 50°C in different pH buffers (3.0–9.0) as mentioned in the text. Activity at pH 4.5 was taken as control (100%) for calculation of remaining percent activity. Enzyme activity was determined as described in the text. Symbols show (●) soluble and (○) immobilized *β*-galactosidase.

**Figure 3:**
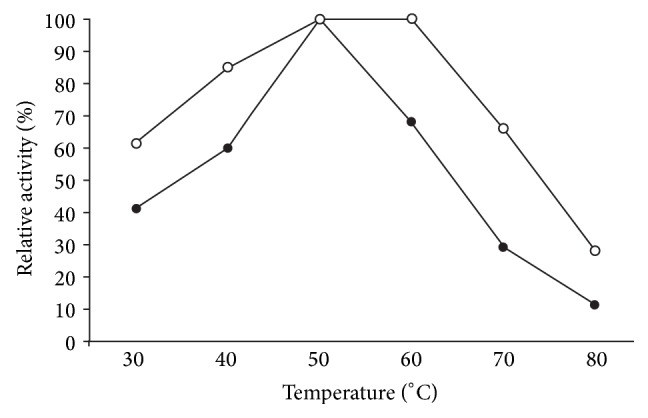
Temperature activity profiles for soluble and CA-PMMA bound *β*-galactosidase. Activity of soluble and immobilized *β*-galactosidase (20 *μ*L) was assayed in 100 mM sodium acetate buffer pH 4.5 at various temperatures (30–80°C) for 15 min. Activity obtained at 50°C was considered as control (100%) for calculation of remaining percent activity for soluble and immobilized enzyme. For symbols, please refer to Figure 2.

**Figure 4:**
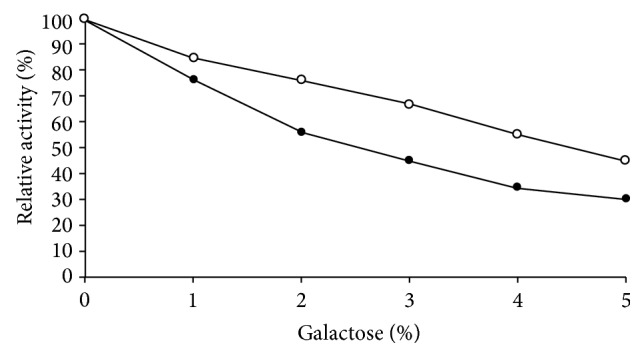
Effect of galactose on soluble and CA-PMMA bound *β*-galactosidase. Effect of galactose on soluble and immobilized *β*-galactosidase (20 *μ*L) was measured in the presence of increasing concentrations of galactose (1.0–5.0%, w/v) in 100 mM sodium acetate buffer pH 4.5 for 1 h at 50°C. Activity of enzyme without added galactose was considered as control (100%) for the calculation of remaining percent activity at other concentrations. For symbols, please refer to Figure 2.

Soluble *β*-galactosidase showed 66% activity at pH 4.0 while I*β*G exhibited 80% activity under identical conditions (Figure 2). Moreover, temperature-optimum was broadened from 50 to 60°C for the immobilized enzyme (Figure 3).  Figure 4 demonstrated that immobilized *β*-galactosidase showed greater resistance to product inhibition mediated by galactose as compared to the soluble counterpart. It was observed that S*β*G showed 56% and 35% activity at 2.0% and 4.0% galactose concentration while the immobilized enzyme retained 76% and 55% activity under identical incubation conditions. Marked increase in stability of immobilized *β*-galactosidase under various denaturing conditions reflected the conformational stability attained by enzyme as a result of bond formation between enzyme and matrix, and lower restriction to substrate diffusion [12, 13].

### 3.4. Lactose Hydrolysis from Milk and Whey in Batch Process

Hydrolysis of lactose from milk and whey was obtained by lab-scale batch reactors operated at 50°C for 10 hours (Table 3). It was observed that greater percent of lactose was hydrolyzed by S*β*G during initial hours as compared to I*β*G. It was due to the fact that soluble enzyme was more accessible for the hydrolysis of lactose during initial few hours. However, after prolonged incubation, rate of lactolysis decreased much faster for soluble enzyme as compared to immobilized *β*-galactosidase due to greater inhibition of soluble enzyme by galactose [9]. The result showed that after 4 hours S*β*G showed 66% and 59% lactose hydrolysis from whey and milk, respectively, while I*β*G exhibited 75% and 69% activity under similar conditions. Moreover, the maximum lactolytic activity obtained for whey and milk by S*β*G was 75% and 66%, respectively, while for immobilized enzyme, the corresponding values were 87% and 83%, respectively. I*β*G exhibited greater efficiency in hydrolyzing lactose from whey as compared to milk lactose because of the difference observed in pH between the tests, that is, pH 4.5–5.0 for whey and pH 6.5–6.8 for milk. It has been reported by previous investigators that* Aspergillus oryzae* *β*-galactosidase showed 100% activity at pH 4.5 but its activity considerably decreased above pH 6.0 [13, 21].

### 3.5. Stability Studies of Immobilized *β*-Galactosidase

**Figure 5:**
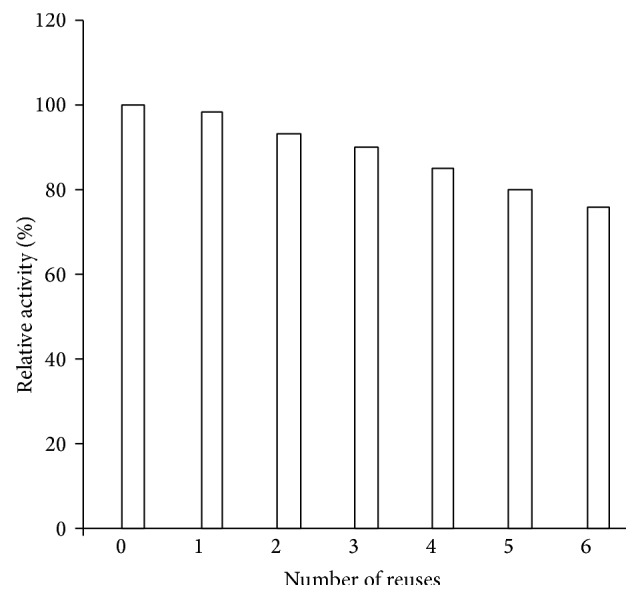
Reusability of CA-PMMA bound *β*-galactosidase. Reusability of immobilized *β*-galactosidase was monitored for 6 successive days. The preparation was taken in triplicates and was assayed for remaining percent activity. Activity determined on the first day was taken as control (100%) for calculation of remaining activity after each use.

**Figure 6:**
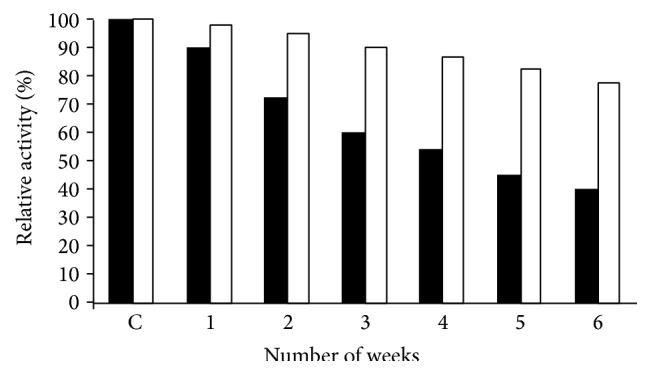
Storage stability of soluble and CA-PMMA bound *β*-galactosidase. Soluble and CA-PMMA bound *β*-galactosidase was stored in 100 mM sodium acetate buffer pH 4.5 at 4°C for 6 weeks. The aliquots from each preparation (20 *μ*L) were taken in triplicates at the gap of 1 week and were then analyzed for the remaining enzyme activity.

I*β*G exhibited 86% activity even after its fifth repeated use (Figure 5). Moreover, it showed 78% of the initial enzyme activity after 6 weeks of storage at 4°C as compared to the retainment of 40% activity by the soluble *β*-galactosidase under identical conditions (Figure 6). The generally observed higher stability of I*β*G upon repeated use and storage is related to the specific and strong binding of enzyme with the support which prevented the unfolding/denaturation of enzyme upon long storage [12, 24, 25].

## 4. Conclusion

Cellulose acetate-polymethylmethacrylate may prove to be an important matrix for immobilizing other industrially important enzymes due to its low-cost, large surface area, and less diffusion limitation provided in transporting substrate and product for enzymatic reactions. In view of its stability and utility against various physical and chemical denaturants and in batch process, such preparation could be exploited for the continuous conversion of lactose from milk and whey for longer durations in a reactor in a more convenient and cheaper way.

## Figures and Tables

**Table 1:** *β*-Galactosidase immobilized on CA-PMMA.

Enzyme activity loaded (*X* Units)	Enzyme activity in washes (*Y* Units)	Activity bound on CA-PMMA		Activity yield (%) *B*/*A* × 100
		Theoretical (*X* − *Y*) = *A*	Actual = *B*	
15000	1256	13744	12782	93

Each value represents the mean for three independent experiments performed in triplicates, with average standard deviations, <5%.

**Table 2:** Kinetic parameters for soluble *β*-galactosidase and enzyme immobilized on CA-PMMA.

Enzyme preparation	Michaelis-Menten constant (mM)	Maximum reaction rate (mM/min)
S*β*G	3.56 ± 1.4	2.76 ± 1.7
I*β*G	3.88 ± 1.8	1.93 ± 1.3

Each value represents the mean for three independent experiments performed in triplicates, with average standard deviations, <5%.

**Table 3:** Hydrolysis of lactose from milk and whey by soluble and immobilized *β*-galactosidase in batch process at 50°C.

Time (h)	Whey		Milk	
	S*β*G	I*β*G	S*β*G	I*β*G
Control	0	0	0	0
1	53 ± 1.9	42 ± 2.7	35 ± 1.8	31 ± 1.8
2	57 ± 2.2	61 ± 2.9	41 ± 2.1	48 ± 3.7
3	62 ± 1.8	67 ± 3.8	46 ± 1.9	62 ± 2.8
4	66 ± 2.7	75 ± 3.9	59 ± 3.4	69 ± 3.4
5	67 ± 3.6	79 ± 1.9	60 ± 2.6	75 ± 1.8
6	71 ± 1.8	82 ± 1.4	62 ± 3.2	79 ± 3.8
7	73 ± 2.8	84 ± 2.2	62 ± 2.9	80 ± 4.2
8	75 ± 3.2	85 ± 2.7	64 ± 2.1	80 ± 3.2
9	75 ± 3.5	87 ± 2.8	66 ± 1.9	83 ± 2.5
10	75 ± 2.3	87 ± 3.6	66 ± 3.7	83 ± 2.9

Numeric values represent the percentage of lactose hydrolyzed from milk and whey at indicated time interval. Each value represents the mean for three independent experiments performed in triplicates, with average standard deviations, <5%.

## Abbreviations

CA-PMMA: Cellulose acetate-polymethylmethacrylate
I*β*G: Immobilized *β*-galactosidase
S*β*G: Soluble *β*-galactosidase
ONPG: * o*-Nitrophenyl *β*-D-galactopyranoside.
